# Multiomics Differences in Lung Squamous Cell Carcinoma Patients with High Radiosensitivity Index Compared with Those with Low Radiosensitivity Index

**DOI:** 10.1155/2021/3766659

**Published:** 2021-08-30

**Authors:** Yajing Du, Sujuan Yuan, Xibing Zhuang, Qi Zhang, Tiankui Qiao

**Affiliations:** Center for Tumor Diagnosis and Therapy, Jinshan Hospital, Shanghai 201508, China

## Abstract

**Objectives:**

Radiosensitivity Index (RSI) can predict intrinsic radiotherapy sensitivity. We analyzed multiomics characteristics in lung squamous cell carcinoma between high and low RSI groups, which may help understand the underlying molecular mechanism of radiosensitivity and guide optional treatment for patients in the future.

**Methods:**

The Cancer Genome Atlas (TCGA) and the Gene Expression Omnibus (GEO) data were used to download clinical data, mRNA, microRNA, and lncRNA expression. Differential analyses, including mRNA, miRNA, lncRNA, and G.O. and KEGG, and GSVA analyses, were performed with R. Gene set enrichment analysis was done by GSEA. miRNA-differentially expressed gene network and ceRNA network were analyzed and graphed by the Cytoscape software.

**Results:**

In TCGA data, 542 patients were obtained, including 171 in the low RSI group (LRSI) and 371 in the high RSI group (HRSI). In RNAseq, 558 significantly differentially expressed genes (DEGs) were obtained. KRT6A was the most significantly upregulated gene and IDO1 was the most significantly downregulated gene. In miRNAseq, miR-1269a was the most significantly upregulated. In lncRNAseq, LINC01871 was the most upregulated. A 66-pair interaction between differentially expressed genes and miRNAs and an 11-pair interaction between differential lncRNAs and miRNAs consisted of a ceRNA network, of which miR-184 and miR-490-3p were located in the center. In the GEO data, there were 40 DEGs. A total of 17 genes were founded in both databases, such as ADAM23, AHNAK2, BST2, COL11A1, CXCL13, FBN2, IFI27, IFI44L, MAGEA6, and PTGR1. GSVA analysis revealed 31 significant pathways. GSEA found 87 gene sets enriched in HRSI and 91 gene sets in LRSI. G.O. and KEGG of RNA expression levels revealed that these genes were most enriched in T cell activation and cytokine−cytokine receptor interaction.

**Conclusions:**

Patients with lung squamous cell carcinoma have different multiomics characteristics between two groups. These differences may have an essential significance with radiotherapy effect.

## 1. Introduction

Lung cancer, the first killer globally, was estimated at 131,880 deaths in 2021 [1]. Lung squamous cell carcinoma (LUSC) accounts for 20–30% of NSCLCs [2].

Radiotherapy is one of the effective cancer treatments. Radiosensitivity Index (RSI) is a novel model of tumor radiosensitivity. Based on the expression of 10 genes (JUN, STAT1, SUMO1, IRF1, HDAC9, ABL1, CDK1, RELA, PRRT2, and AR), RSI could predict intrinsic radiotherapy sensitivity and treatment response [3]. This model is widely used in cancer, such as breast cancer and NSCLCs [4–6].

Our study analyzed mRNA, miRNA, lncRNA, methylation, somatic mutations, copy number variations, and clinical data between high RSI and low RSI groups in LUSC patients. This research may reference precision radiotherapy research and help build personalized precision management of patients in clinical applications.

## 2. Material

### 2.1. TCGA Data

The data were downloaded from The Cancer Genome Atlas (TCGA) data portal (https://portal.gdc.cancer.gov/)(TCGA-LUSC) through https://xenabrowser.net/datapages/, including mRNA and miRNA expression data, methylation array, mutation profiles, copy number variation, and clinical data [7]. After matching clinical data, 363 cases of mRNA, 542 instances of miRNA, 542 cases of *lnc*RNA, 362 cases of DNA methylation, 480 cases of somatic mutation, and 490 cases of copy number variation were selected for further analysis between the RSI high score group (HRSI) and the low score group (LRSI).

### 2.2. GEO Data

mRNA data was downloaded from the Gene Expression Omnibus (GEO) database (http://www.ncbi.nlm.nih.gov/geo/).

GSE73403 and GSE37745 datasets were collected for the differential gene expression analysis. GSE73403 dataset contains 69 samples from the LUSC patients, published on Sep 25, 2015, and GSE37745 includes 66 samples, released on Oct 12, 2012.

## 3. Statistical Methods

### 3.1. Statistics of Group

According to previous studies, ten genes (JUN, STAT1, SUMO1, IRF1, HDAC9, ABL1, CDK1, RELA, PRRT2, and AR) were picked out for each sample to calculate RSI (Radiosensitivity Index). The equation is as follows:

RSI = −0.0098009∗AR + 0.0128283∗JUN + 0.0254552∗STAT1 − 0.0017589∗PRRT2 − 0.0038171∗RELA + 0.1070213∗ABL1 − 0.0002509∗SUMO1 − 0.0092431∗CDK1 − 0.0204469∗HDAC9 − 0.0441683∗IRF1.

The R software (version 4.0.0) was applied to statistical analyses. Cutpoint of RSI was performed by the survminer package of R with the function of surv_cutpoint, which was design to determine the optimal cutpoint for continuous variables.

### 3.2. Differential mRNA, miRNAs, lncRNAs, and ceRNA Analysis

In TCGA dataset, after normalization, differential gene analyses, including mRNAseq, miRNAseq, and lncRNAseq, were done by the R limma package. For mRNAseq, the absolute logfoldchange (∣logFC∣) > 0.5 and the adjusted *p* < 0.05 were considered to be significant. For miRNAseq, ∣logFC | >0.5 and *p* < 0.05 were significant in statistics science. As for lncRNAseq, ∣logFC | >0.25 and adjusted *p* < 0.05 were statistically significant. For mRNAseq from the GEO dataset, ∣logFC | >0.5 and the *p* < 0.05 were statistically significant.

Differential lncRNAs targeted miRNAs were achieved through http://mircode.org/index.php.

Differential miRNAs targeted mRNAs were achieved through http://mirwalk.umm.uni-heidelberg.de/search_mirnas. These data and differential mRNAs were intersected, consisting of a ceRNA network. The Cytoscape software (version 3.7.1) was used to analyze and graph a miRNA-differentially expressed gene network and ceRNA network.

Gene Ontology (G.O.) and Kyoto Encyclopedia of Genes and Genomes (KEGG) were performed by the ClusterProfiler package for the mRNAs, miRNAs, and lncRNAs between HRSI and LRSI patients. Gene set variation analysis was done by the GSVA package. Gene set enrichment (GSEA) was carried out by GSEA (version 4.0.0).

### 3.3. Copy Number Variation and Somatic Mutation Analysis

Significantly mutated genes, pfamDomains were done by the maftools package of R. The threshold for significant mutated genes, pfamDomains was *p* < 0.05.

### 3.4. DNA Methylation Analysis

Differentially methylated regions, differentially methylated positions, and differentially methylated gene analyses were performed by the minif package. *p* < 0.05 was considered statistically significant for methylated genes, while adjusted *p* < 0.05 was for methylated regions. In differentially methylated positions, the adjusted *p* < 0.05 was considered to be statistically significant.

Differentially methylated genes and differentially expressed genes were jointly analyzed to find methylation driver genes.

## 4. Results

### 4.1. Clinical Characteristics and Survival Analyses

**Figure 1:**
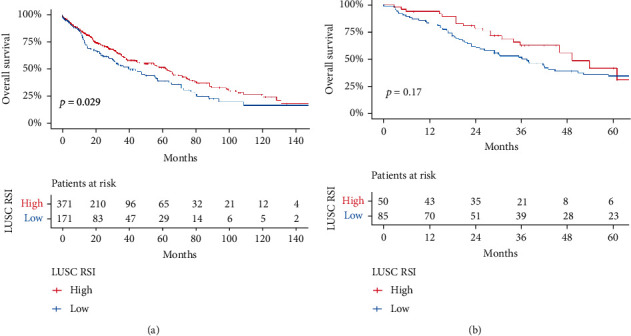
(a) Survival analysis of high and low RSI groups in TCGA dataset. (b) Survival analysis of high and low RSI groups in the GEO dataset.

In TCGA data, based on RSI scores (0.50 tangents), 542 LUSC patients were divided into RSI high grouping (HRSI) and low grouping (LRSI), of which 171 were in LRSI and 371 were in HRSI. The results showed that there was an obvious survival difference between HRSI and LRSI (*p* = 0.029). In two GEO datasets, the cutpoint of RSI was 0.54 and 0.55. We merged two GEO datasets and found the survival of patients with high RSI scores in the GEO database was still better than those with low scores, though not significant (*p* = 0.17) but possibly due to the small number of the patients (50 patients in HRSI vs. 85 patients in LRSI) (Figures 1(a) and 1(b)). Clinical characteristics of patients in the HRSI and LRSI groups are shown in Tables 1 and 2.

### 4.2. Differentially Expressed Genes, miRNAs, lncRNAs, and ceRNA Network

**Figure 2:**
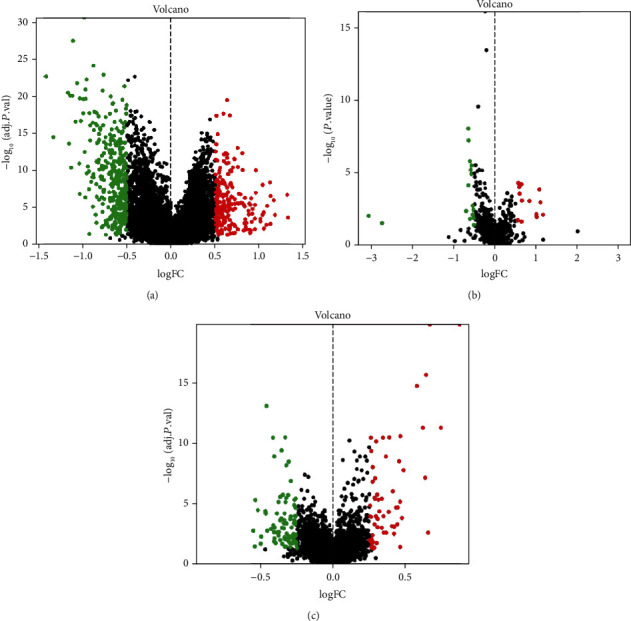
(a) Volcano map of differential expression mRNAs, (b) volcano map of differential expression miRNAs, and (c) volcano map of differential expression lncRNAs.

The low RSI group was used as a reference in the analyses. In RNAseq, 558 significantly differentially expressed genes (DEGs) were obtained; 334 were upregulated, and 224 were downregulated (Figure 2(a)). KRT6A and IDO1 were the most significantly upregulated (logFC = 1.32, adj.*p* = 0.0002) and downregulated (logFC = −1.42, adj.*p* < 0.0001) genes, respectively.

In the GEO database, there were 12 upregulated genes and 28 downregulated genes. FBN2 and MAGEA6 were the most significantly upregulated (logFC = 0.85, *p* value = 0.001) and downregulated (logFC = −1.02, *p* value = 0.01) genes, respectively.

After intersecting the DEGs in the two databases, we found the total of 17 genes in both databases, including ADAM23, AHNAK2, BST2, COL11A1, CXCL10, CXCL11, CXCL13, FBN2, HAS3, IFI27, IFI44L, IFIT1, IFIT3, MAGEA6, MMP13, NEFL, and PTGR1.

In TCGA database, 31 differentially expressed miRNAs (DEMs) were obtained. (Figure 2(a)). miR-1269a was the most significantly upregulated (logFC = 1.17, *p* = 0.0089), while miR-875-3p was the most significantly downregulated (logFC = −3.06, *p* = 0.0089). And in lncRNAseq (Figure 2(c)), the number of differentially expressed lncRNAs (DELs) was 145, in which LINC01871 was the most significantly upregulated (logFC = 0.87, adj.*p* < 0.0001) and AL049555.1 was the most significantly downregulated (logFC = −0.55, adj.*p* = 0.0016).

**Figure 3:**
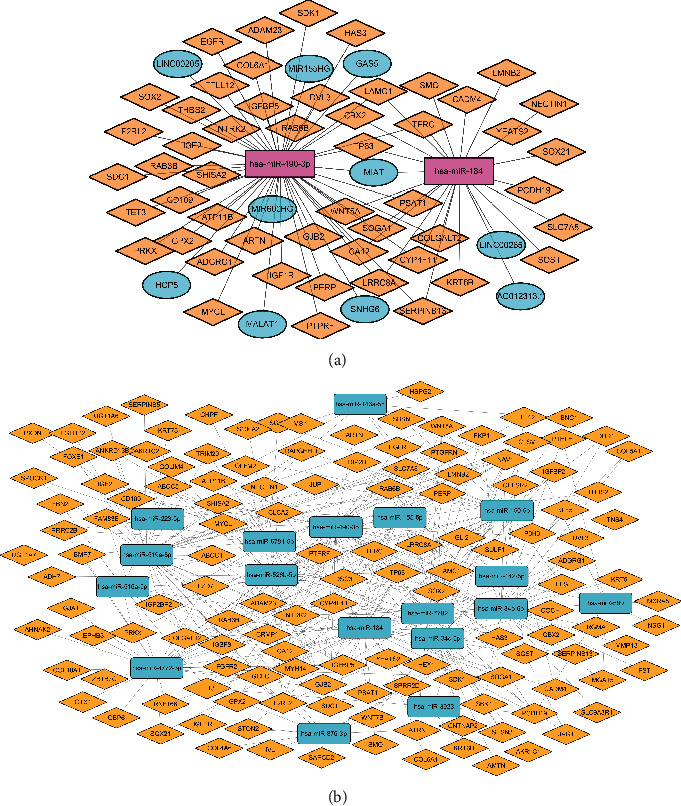
(a) MicroRNAs-differentially expressed gene (miRNA-DEG) pairs. (b) ceRNA network of differential lncRNA-differential miRNA-differentially expressed gene (miRNA-DEG and lncRNA-DEG) pairs.

We used a website http://mirwalk.umm.uni-heidelberg.de/search_mirnas/, predicting miRNAs' target genes, and intersected with the DEGs to draw miRNA-target maps. A 66-pair interaction between differentially expressed genes and miRNAs and an 11-pair interaction between differentially lncRNAs and miRNAs consisted of a ceRNA network, of which miR-184 and miR-490-3p were located in the center. These miRNAs may play critical roles in radiosensitivity (Figures 3(a) and 3(b)).

### 4.3. Functional Analysis

**Figure 4:**
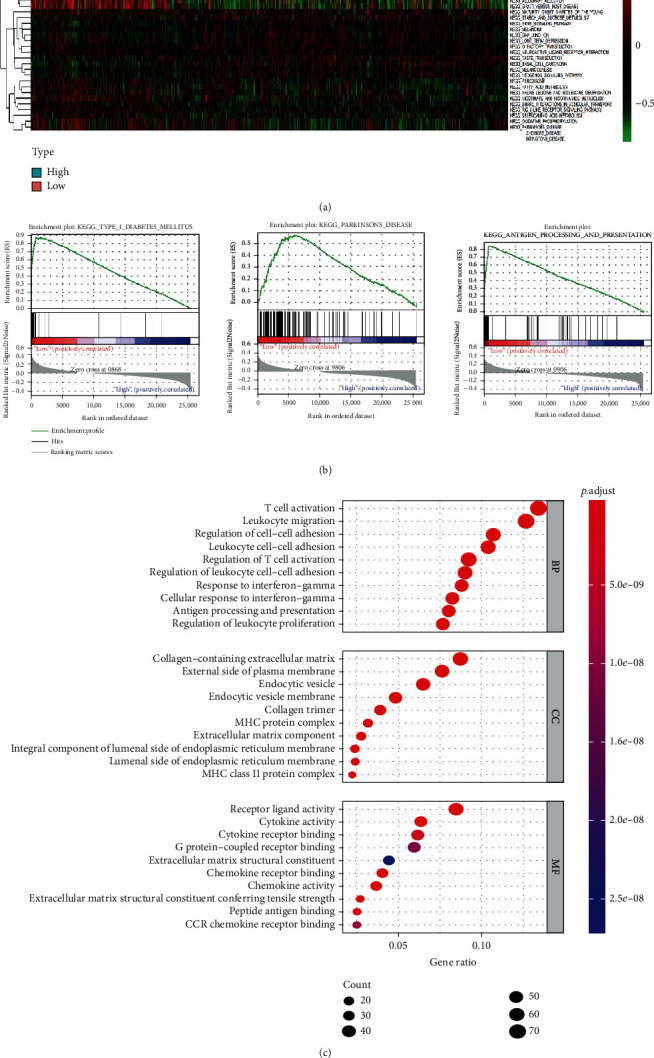
(a) Heatmap of gene set variation analysis for GSVA. (b) The three most significant pathways of GSEA. (c) Dotplot of significantly different pathways from G.O.

GSVA analysis revealed 31 significant pathways, including hedgehog_signaling_pathway, erbb_signaling_pathway, and apoptosis. GSEA found that 87 gene sets were enriched in the HRSI group, including hedgehog_signaling_pathway, while 91 gene sets were enriched in the LRSI group, including natural_killer_cell_mediated_cytotoxicity, toll_like_receptor_signaling_pathway, and cytosolic_DNA_sensing_pathway (Figures 4(a) and 4(b)).

G.O. and KEGG of RNA expression levels revealed that these genes were most significantly enriched in T cell activation and cytokine−cytokine receptor interaction (Figure 4(c)).

### 4.4. SNV and CNV Analysis

**Figure 5:**
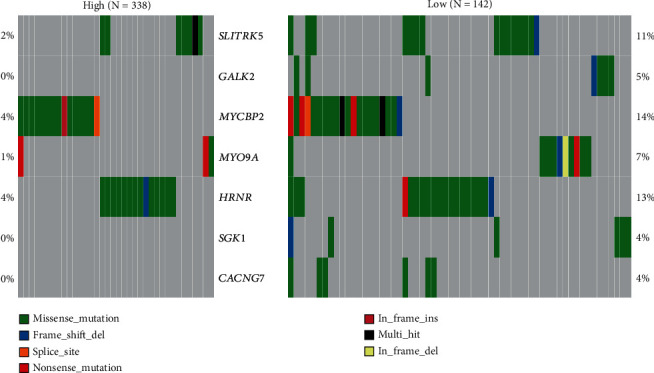
Somatic mutation waterfall map of the most significant genes between HRSI and LRSI.

In 480 LUSC patients, the mutation proportion of the most significant genes (SLITRK5, GALK2, MYCBP2, MYO9A, HRNR, SGK1, and CACNG7) between the two groups of HRSI (*n* = 338) and LRSI (*n* = 142) is shown in Figure 5.

**Figure 6:**
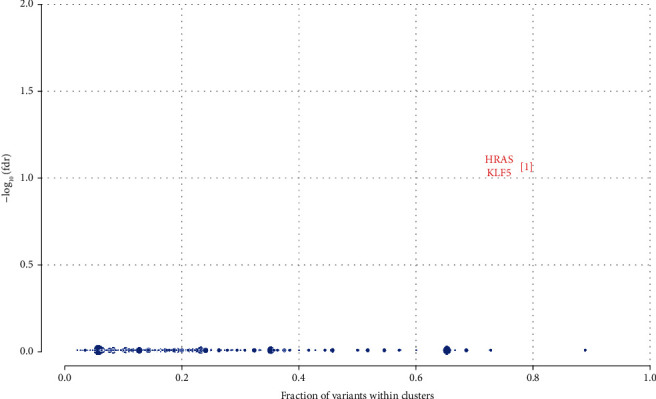
Cancer-driven mutations in the HRSI group.

**Figure 7:**
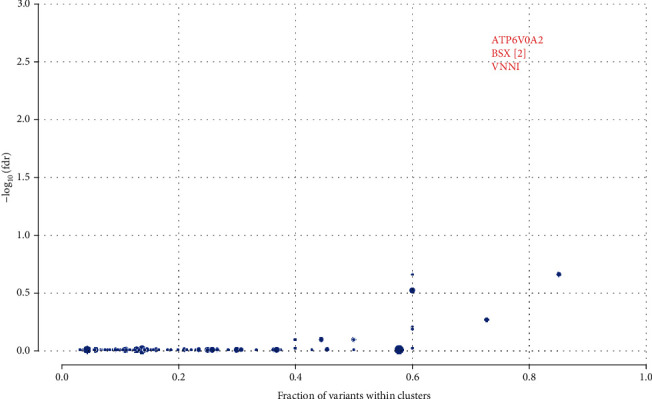
Cancer-driven mutations in the LRSI group.

After using the mafCompare function, we obtained 212 differential mutation genes, and most of the differential mutation genes in the LRSI group had higher mutation rates. In terms of cancer-driven mutations, the HRSI group has two significant mutations, including HRAS and KLF5, while the LRSI group has three significant mutations, including ATP6V0A2, BSX, and VNN1 (Figures 6 and 7).

**Figure 8:**
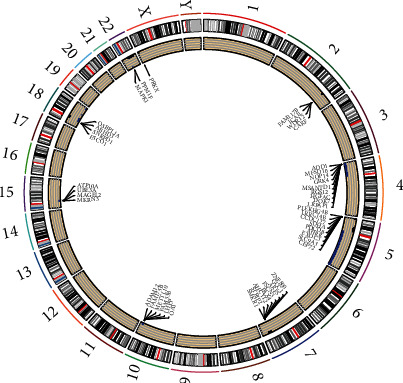
The differences in CNV between the two groups. The black dots indicate the copy number amplified regions, and the blue dots indicate the copy number deletion regions in the HRSI group.

We analyzed changes in chromosomal regions in two groups. There are statistical differences between 876 deletion fragments and 239 amplification fragments (*p* < 0.05). MN1 was the most significant amplification fragments (*p* = 0.004), and SGCD was the deletion fragments (*p* = 0.0007). Most of amplification regions were located on chromosome 2, 5, 7, 18, and 22, while most of the deletion regions were on chromosomes 4, 5, 10, 15, 18, and X. As is shown in the figure, in the HRSI group, deletion regions were most evident on chromosome 5 (Figure 8).

### 4.5. DNA Methylation Analysis

**Figure 9:**
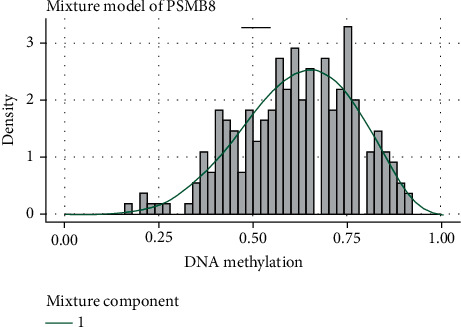
A PSMB8 methylation level distribution curve.

**Figure 10:**
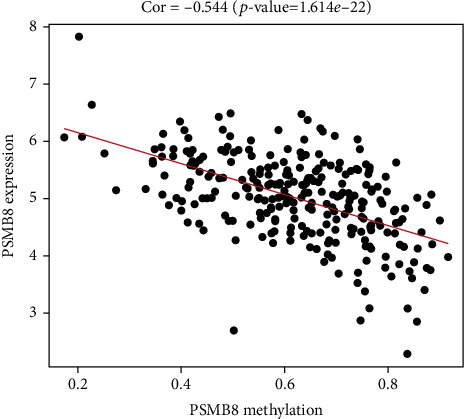
Relationship between PSMB8 methylation level and gene expression.

After quality control, there were 35 upregulated and 231 downregulated methylation positions detected in the HRSI group. Then, we analyzed the differential methylation regions (DMRs). 70 DMRs were obtained, and we used the DMRs to annotate the functional consequences of genetic variation through http://wannovar.wglab.org/. It showed that the most significant DMR was ZFP36L2. We analyzed the methylation genes and mRNAs to obtain methylation-driven genes in the HRSI group. It showed a total of 8 significant genes, including PSMB8, AIM2, GBP4, ACSL5, CD74, OAS2, TRAF2, and ZBTB24, in which PSMB8 is the most significant driven gene (Figures 9 and 10).

## 5. Discussion

Personalized and precise treatment of cancer patients is a medicine goal at present. RSI is of great significance to the individualization of tumor radiation therapy.

Our study used TCGA and GEO databases to determine the relationship between RSI score and multiomics genetic differences in LUSC patients. In differentially expressed mRNA analyses, we found gene expression, such as KRT6A and IDO1, is the most significant mRNAs. As is shown in the results, compared with the LRSI group, IDO1 expression in the HRSI group was upregulated. The level of gene transcription of IDO1 is closely related to T cell infiltration [8–12]. In some studies, IDO1 enzymatic activity can directly influence radiation sensitivity, such as colorectal cancer [13–17]. We found some genes in TCGA and GEO databases are related to radiation therapy, including FBN2, IFI27, and IFIT1. Forrester et al. found that FBN2 was associated with radiation-induced fibrosis [18]. STAT1, associated with increased resistance to radiation, regulated IFI27 and IFIT1, which indicated that IFI27 and IFIT1 might be involved in radiation sensitivity.

In differentially expressed miRNA analyses, miR-1269a is the most significant miRNAs. miR-1269a is significantly more expressed in NSCLC tissue than in adjacent tissue. miR-1269a expression upregulation enhances cell proliferation and cluster formation and induces cell cycle conversion. miR-1269a could function as an onco-miRNA in NSCLC and promote NSCLC growth via downregulating SOX6 [19]. SOX6 suppresses the cell cycle of lung adenocarcinoma by regulating cyclin D1, which indicated miR-1269a might be involved in radiation sensitivity [20]. In lncRNA analyses, LINC01871 was the most significant one. LINC01871 was related to the immunotherapeutic strategy and was used to predict the prognosis of patients with cervical cancer. Radiotherapy combined with immunity will be the next oncology practice [21].

We enriched the function of genes, and the results showed that the upregulated gene pathways in the HRSI group have a stronger relationship with cytosolic_DNA_sensing_pathway. That implied that differences might relate to the sensitivity of radiotherapy.

In SNV and CNV analysis, the mutation proportion of the most significant genes included SLITRK5, SGK1, and CACNG7. SLITRK5 involved radioresistance in nasopharyngeal carcinoma [22]. Several studies have shown that SGK1 can increase radiotherapy sensitivity through various means, in multiple cancers, such as lung cancer, glioblastoma, and synovial sarcoma [22–29].

In DNA methylation analyses, ZFP36L2 is the most significant differential methylation region. A recent study shows that ZFP36L2 inhibited cell proliferation through the cell cycle, which implied that it might be involved in radiation sensitivity [30]. Immune proteasome (PSMB8) is the most significant regional methylation gene between the two groups. PSMB8 is associated with proliferation and apoptosis and is considered a novel prognostic indicator in patients [31]. Ha et al. thought PSMB8 was a predictive marker of preoperative radiosensitivity [32]. Compared with the LRSI group, KLF5 was the most significant gene for CNV in the HRSI group. KLF5 plays an important role in the DNA damage response by regulating DNA damage checkpoint proteins and is associated with cisplatin DDP resistance [33–35]. The relationship between KLF5 and radiotherapy sensitivity needs to be discovered.

Finally, there are some flaws in our experiment. Because the GEO database lacks methylation datasets, we cannot use GEO data to compare methylation mutations. There is also a lack of clinical samples for radiation therapy, so we cannot verify that the differences we find are related to radiation sensitivity.

## 6. Conclusion

In summary, our study used TCGA and GEO data to investigate multicomponent differences between patients with LUSC high and low RSI. Our research can refer to precision radiotherapy studies and help build personalized precision management for patients in clinical applications.

## Figures and Tables

**Table 1:** Clinical characteristics of TCGA samples.

Characteristics	RSI high expression group (*n* = 368)		RSI low expression group (*n* = 170)		*p* value
Age	67.44		67.08		0.65
Sex					
Female	95	(25.8)	46	(27.1)	0.842
Male	273	(74.2)	124	(72.9)	
Anatomic location					
Bronchial	6	(1.6)	4	(2.4)	0.755
Left	155	(42.1)	68	(40.0)	
Right	197	(53.5)	91	(53.5)	
Other (please specify)	7	(1.9)	3	(1.8)	
NA	2	(0.5)	3	(1.8)	
Discrepancy					
Stage					
Stage I	180	(48.9)	85	(50.0)	0.252
Stage II	125	(34.0)	48	(28.2)	
Stage III	57	(15.5)	31	(18.2)	
Stage IV	5	(1.4)	3	(1.8)	
Discrepancy	1	(0.3)	3	(1.8)	
Histological type					
LUSC	349	(94.8)	165	(97.1)	0.325
Basaloid LUSC	12	(3.3)	4	(2.4)	
Papillary LUSC	6	(1.6)	0	(0.0)	
Small cell LUSC	1	(0.3)	1	(0.6)	

**Table 2:** Clinical characteristics of the GEO samples.

Characteristics	RSI high expression group (*n* = 50)		RSI low expression group (*n* = 85)		*p* value
Age	61.28		63.66		0.166
Sex					
Female	1	(2.0)	23	(27.1)	0.001
Male	49	(98.0)	62	(72.9)	
Stage					
Stage I	7	(14.0)	36	(42.4)	0.001
Stage I or stage II	14	(28.0)	8	(9.4)	
Stage II	16	(32.0)	20	(23.5)	
Stage III	13	(26.0)	21	(24.7)	

## Data Availability

TCGA data: the data were downloaded from The Cancer Genome Atlas (TCGA) data portal (https://portal.gdc.cancer.gov/)(TCGA-LUSC) through https://xenabrowser.net/datapages/, including mRNA and miRNA expression data, methylation array, mutation profiles, copy number variation, and clinical data. After matching clinical data, 363 cases of mRNA, 542 instances of miRNA, 542 cases of lncRNA, 362 cases of DNA methylation, 480 cases of somatic mutation, and 490 cases of copy number variation were selected for further analysis between the RSI high score group (HRSI) and the low score group (LRSI). GEO data: mRNA data was downloaded from the Gene Expression Omnibus (GEO) database (http://www.ncbi.nlm.nih.gov/geo/). GSE73403 and GSE37745 datasets were collected for the differential gene expression analysis. GSE73403 dataset contains 69 samples from the LUSC patients, published on Sep 25, 2015, and GSE37745 includes 66 samples, released on Oct 12, 2012.
